# Assessing the translatability of In vivo cardiotoxicity mechanisms to In vitro models using causal reasoning

**DOI:** 10.1186/2050-6511-14-46

**Published:** 2013-09-06

**Authors:** Ahmed E Enayetallah, Dinesh Puppala, Daniel Ziemek, James E Fischer, Sheila Kantesaria, Mathew T Pletcher

**Affiliations:** 1 Compound Safety Prediction, Pfizer Inc., Groton, CT, USA; 2 Drug Safety Research & Development, Pfizer Inc., Groton, CT, USA; 3 Computational Sciences CoE, Pfizer Inc., Cambridge, MA, USA; 4 Rare Disease Research Unit, Pfizer Inc., Cambridge, MA, USA

**Keywords:** Causal reasoning, Cardiotoxicity, Translatability, In vitro screening, Preclinical safety

## Abstract

Drug-induced cardiac toxicity has been implicated in 31% of drug withdrawals in the USA. The fact that the risk for cardiac-related adverse events goes undetected in preclinical studies for so many drugs underscores the need for better, more predictive in vitro safety screens to be deployed early in the drug discovery process. Unfortunately, many questions remain about the ability to accurately translate findings from simple cellular systems to the mechanisms that drive toxicity in the complex in vivo environment. In this study, we analyzed translatability of cardiotoxic effects for a diverse set of drugs from rodents to two different cell systems (rat heart tissue-derived cells (H9C2) and primary rat cardiomyocytes (RCM)) based on their transcriptional response. To unravel the altered pathway, we applied a novel computational systems biology approach, the Causal Reasoning Engine (CRE), to infer upstream molecular events causing the observed gene expression changes. By cross-referencing the cardiotoxicity annotations with the pathway analysis, we found evidence of mechanistic convergence towards common molecular mechanisms regardless of the cardiotoxic phenotype. We also experimentally verified two specific molecular hypotheses that translated well from in vivo to in vitro (Kruppel-like factor 4, *KLF4* and Transforming growth factor beta 1, *TGFB1*) supporting the validity of the predictions of the computational pathway analysis. In conclusion, this work demonstrates the use of a novel systems biology approach to predict mechanisms of toxicity such as KLF4 and TGFB1 that translate from in vivo to in vitro. We also show that more complex in vitro models such as primary rat cardiomyocytes may not offer any advantage over simpler models such as immortalized H9C2 cells in terms of translatability to in vivo effects if we consider the right endpoints for the model. Further assessment and validation of the generated molecular hypotheses would greatly enhance our ability to design predictive in vitro cardiotoxicity assays.

## Background

In 2007, the leading cause for drug withdrawal from the market was attributed to cardiotoxicity (31%) [1]. The voluntary withdrawal of the COX-2 selective inhibitor Rofecoxib in 2004 due to increased risk of myocardial infarction and stroke is one of the more prominent examples [2]. Addressing the safety issues early would significantly reduce such costly surprises in the drug discovery process and would also improve the survival of pharmaceutical drugs to the market. Although using animal models to predict late stage safety issues has been the norm in the industry for years, there is increased expectation that progress in utilization of computational toxicology predictive models, specialized in vitro models and a combination of both these models will enhance early de-risking, reduce animal use and enhance compound survival. In addition, the US National Academy of Sciences recently released a toxicity testing framework emphasizing the utilization of high throughput in vitro toxicity assays and computational models to assess the risk and underlying mechanism of toxicities triggered by pharmaceutical chemicals and environmental contaminants. This is envisioned to include model systems based on stem cell biology, functional genomics and physiologically based pharmacokinetic (PBPK) modeling [3].

There have been several reports wherein computational models have been utilized for predicting the early safety risks based on potassium voltage-gated channel, subfamily H (HERG) binding [4,5], Absorption, Distribution, Metabolism, Excretion and Toxicity (ADMET) properties [6], Adenosine tri-phosphate Binding Cassette (ABC) transporter substrates [7] and Cytochrome P450 (CYP450) inductions [8]. However, the successful utilization of mechanism-based screening assays has been a challenge despite the plethora of published studies on the known mechanisms of drug-induced cardiac toxicity. These include well studied mechanisms of cardiotoxicity such as oxidative stress, calcium dysregulation, energy metabolism disruption, cell cycle/proliferation and tissue remodeling [9-11].

It is believed that a major factor contributing to the limited success of predicting clinical outcome using preclinical models or predicting in vivo outcome using in vitro models is due to limited understanding of the translatability across model systems and species. Hence, the recent increase of models believed to better reflect the physiological and functional roles of cardiomyocytes such as progenitor cardiomyocytes, human embryonic stem cells (ESC) and inducible pluripotent stem cell (iPS) derived cardiomyocytes [12,13]. Recently, Force and Kolaja reviewed the most commonly used models of cardiomyocytes summarizing their advantages and disadvantages [11]. It should be noted, of course, that this methodology will only reveal mechanisms that result from direct action of a compound on a cardiomyocyte. This in vitro system is inadequate for predicting secondary effects mediated by the interaction of multiple complex organ systems, such a rise in heart rate due to increased epinephrine release.

The primary goal of this study is to evaluate the translatability of cardiotoxicity mechanisms from in vitro to in vivo and to compare the elicited mechanisms in different in vitro models. To achieve this we utilized gene expression microarray experiments from rat toxicity studies (Drugmatrix, Iconix [14,15]) and in vitro experiments in H9C2 (embryonic BD1X rat heart tissue derived cells) and neonatal rat ventricular cardiomyocytes (RCM) using nine known pharmaceutical compounds known to induce cardiotoxicity in vivo.

The gene expression microarray data was analyzed using a novel computational tool called the Causal Reasoning Engine (CRE) [16,17]. CRE interrogates prior biological knowledge to generate testable hypotheses about the molecular upstream causes of the observed gene expression changes. Each such hypothesis summarizes (“explains”) a certain number of gene expression changes (see *KLF4+* example below). Notably, hypotheses usually make statements about predicted *protein* abundance or activity changes, e.g. increased or decreased TGFB1 activity. In our experience, CRE hypotheses tend to robustly identify biological phenomena driving gene expression changes and provide several advantages over other gene expression analysis methods [17]. In particular, for the purpose of this study, CRE provided the advantage of better abstracting biological information from gene expression data obtained across different experimental settings (see *Causal Reasoning Convergence* below).

Following the CRE analysis of all individual compound treatments in vitro and in vivo, we compared the hypotheses and the biological processes they compose to assess the translatability of mechanisms from one model system to the other. Subsequently, we experimentally tested *KLF4* and *TGFB1* activities, two of the central molecular hypotheses predicted by CRE, in response to the cardiotoxic compounds used in the CRE analysis using qPCR and reporter assay. Finally, we discuss the implications of our analysis and suggest potential future experiments.

## Methods

### Tissue culture

H9C2 cells (derived from embryonic BD1X rat heart tissue) were purchased from ATCC. H9C2 cells were grown DMEM (Gibco# 11965) with 10% FBS as per manufacturer’s protocol. Neonatal, ventricular Clonetics® Rat Cardiac Myocytes (P1-3) (RCM)(Catalog # R-CM-561) were purchased from Lonza and were grown in RCGM media with supplements as per manufacturer’s protocol.

For ATP depletion assays, H9C2 and RCM’s cells were plated in 96 well plates per the manufacturer’s protocol for 24 hr prior to treatments. For gene expression experiments, H9C2 and RCM cells were plated in 24 well plates per the manufacturer’s protocol for 24 hr prior to adding of treatments.

### Chemicals

All the chemicals (Table 1) were purchased from Sigma Aldrich. Stock solutions and working solutions were prepared by dissolving compounds in DMSO.

**Table 1:** In vitro cytotoxicity phenotype (ATP depletion) and known in vivo cardiac safety liabilities of the test compounds

**Drug**	**ATP depletion IC50 at 48 hrs (μM ± SE)**	**In vivo 5-day treatment (mg/kg)**	**Reported ECG abnormalities & arrhythmia**	**Reported structural cardiotoxicity**	**Primary pharmacology & indication**
Amiodarone	12 ± 1.17	147	Yes	No	Anti-arrhythmic
Amitriptyline	5.7 ± 0.67	160	Yes	Yes	Tricyclic antidepressant
Cyclosporine	2.71 ± 0.92	350	No	Yes	Immunosuppressive
Dexamethasone	>300	1	No	Yes	Glucocorticoid
Dobutamine	22.9 ± 0.83	43	Yes	Yes	β1 agonist, inotropic
Doxorubicin	4.23 ± 0.52	3	Yes	Yes	Cytotoxic Anti-neoplastic
Loratadine	39.5 ± 2.11	2000	Yes	No	Anti-histaminic
Mitoxantrone	1.16 ± 0.47	2	Yes	Yes	Cytotoxic Anti-neoplastic
Terbutaline	>300	130	Yes	Yes	β2 agonist

### ATP depletion assays

ATP depletion measurements were done using The CellTiter-Glo® Luminescent Cell Viability Assay from Promega (Catalog # G7570) per the manufacturer’s protocol. 100 μl per well of reconstituted ATP depletion reagent was added directly to 96 well plate and incubated for 10 minutes on orbital shaker. Luminescence signal was measured using Envison plate reader.

### Microarray gene expression data

RNA was extracted 24 hrs after compound treatment using Qiagen’s RNeasy Mini kit (Catalog # 74104) per the manufacturer’s protocol. Quality and quantity of RNA was assessed using Nanodrop 2000c (A260/280 ratio) from Thermo Fisher Scientific and Agilent RNA analyzer (RIN scores). RNA (n = 2) was submitted to Genelogic for Affymetrix Genechip profiling using Rat Expression Array 230 2.0 chip. The in vivo rat cardiac tissue gene expression comparisons in response to the same compounds (Table 1) used in the in vitro experiments were obtained from the Drugmatrix toxicogenomic database [14,15]. The gene expression data for the effect of Isoprenaline on mouse cardiac tissue was obtained from the public domain (http://www.ncbi.nlm.nih.gov/geo/query/acc.cgi?acc=GSE18801), from a study published by Galindo et al. [18].

For quality control, RNA degradation plots were generated for each CEL file. To assess potential RNA degradation, 3′/5′ ratios and their associated confidence intervals were evaluated [19]. Two techniques were used to distill the probe results into a small number of representative variables; Multidimensional scaling (MDS) [20] and Principal component analysis (PCA). These two techniques were applied to the data before and after Robust Multi-Array Average (RMA) [20] signal processing. During this processing, only the perfect match (PM) probe data were used; the mismatch (MM) probes were not used. To assess differential expression of genes between groups of interest, a common statistical model was applied independently to each probeset. Gene expression for all sample types was analyzed on the log2 scale. Linear models were used to calculate t-statistics, which were subsequently adjusted using the moderated t-statistic procedure [21]. The Benjamini and Hochberg adjustment procedure [22] based on controlling the False Discovery Rate (FDR) was used.

### Causal reasoning engine algorithm

Gene expression changes are analyzed to detect potential upstream regulators as previously described [16,17]. Briefly, the approach relies on a large collection of curated biological statements in the form:

**A** [increases or decreases] **B**, where **A** and **B** are measurable biological entities.

The biological entities can be of different types (e.g. phosphorylated proteins, transcript levels, biological process and compound exposure) and each statement is tied to accessible, peer-reviewed articles. For this work, we licensed approximately 450,000 causal statements from commercial sources (Ingenuity Systems and Selventa).

**Figure 1:**
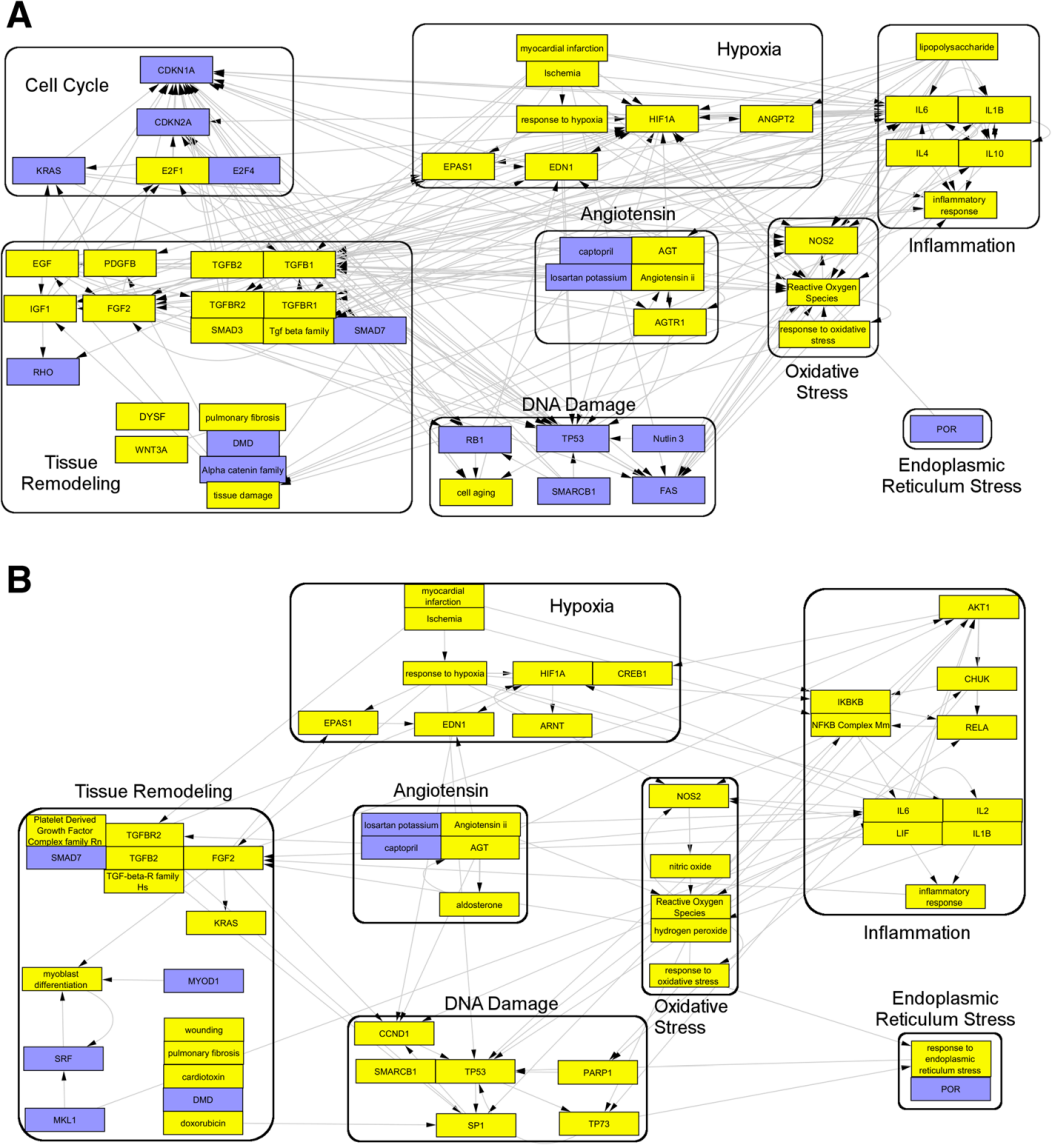
**Ability of CRE to reveal the similar molecular mechanisms of Isoprenaline induced cardiac hypertrophy based on rat (A) and mouse (B) independent experiments.** Both models show similar molecular mechanisms mostly known for their role in hypertrophic cardiomyopathy. (*Blue = predicted decrease, Yellow = predicted increase*).

Each biological entity in the network and its assumed mode of regulation is a potential *hypothesis (*e.g. *predicted decrease in NFE2L2 activity)*. For each hypothesis, we can now compare all possible downstream gene expression changes in the knowledge base with the observed gene expression changes in the experiment. We consider two metrics to quantify the significance of a hypothesis with respect to our experimental data set, namely enrichment and correctness. The *Enrichment* p-value for a hypothesis h quantifies the statistical significance of finding *(#incorrect + #correct)* gene expression changes within the set of all genes downstream of h. The *Correctness* p-value is a measure of significance for the score of a hypothesis h defined as (*#correct - #incorrect*). The *KLF4+* example below shows a depiction of one significant hypothesis with corresponding downstream transcript changes. Molecular entities implicated by individual hypotheses can be grouped into biological processes to get a more comprehensive picture of predicted changes (see example in Figure 1).

### Network modeling of the CRE hypotheses

The analysis results are visualized using the Causal Reasoning Browser, a Java application based on the open-source biological network viewer Cytoscape [23] as previously described [17]. Briefly, in the CRE browser an overview graph allows users to visualize hypotheses and examine their network relationships in the context of the causal relationships obtained from the literature based knowledgebase. To facilitate the construction of biological networks from the generated hypotheses, several analytical tools were developed e.g. a clustering tool uses cosine similarity metric and an average linkage method to group related hypotheses together [24].

### HEK293 TGFβ reporter assay methods

HEK-293 cell line was obtained from American Type Culture Collection (ATCC; Manassas, VA). HEK-293 cells were grown in Eagles Minimum Essential Medium (ATCC) containing 10% fetal bovine serum and 1% penicillin-streptomycin. Cells were maintained at 37°C, 5% CO2, 95% humidity.

TGF*β (SMAD2/SMAD3/SMAD4)* Cignal lentiviral construct and transducing reagents were purchased from SABiosciences (Frederick, MD). Cells were plated in 12-well plates at 2.5×105 cells per well. Transductions were performed according to manufacturer’s directions, using 20 μL of lentiviral particles and 8 μM concentration of Sureentry (SABiosciences) transfection reagent. Stable cell lines were selected using 1 μg/mL puromycin (Sigma, St. Louis, MO). Single cells were isolated from Polyclonal cell lines using a FACS Vantage Cell Sorter (BD, Franklin Lakes, NJ), and expanded.

Transduced cells were plated in 384-well plates at 2000 cells/well. After overnight incubation, cells were induced using 25 ng/ml hTGFβ1 protein (Sigma # T7039) for 1 hour. Cells were then dosed with varying concentrations of test compound at a final 1% DMSO concentration and incubated for 24 hours in a 37° incubator with 5% CO_2_. Luciferase activity was determined using Steady-Glo Luciferase Assay Reagent to cells. (Promega, Madison, WI). Luminescence was measured on an EnVision 2103 Multilabel Reader (Perkin-Elmer, Waltham, MA). To evaluate inhibitory effects of the test compounds on the TGFB1 reporter, it was necessary to first stimulate TGFB1 expression. The in vitro reporter cell lines express low basal levels of TGFB1 by design for the original purpose of agonist evaluation. In addition, the Envision plate reader used for detection of the reporter assay luciferase readout is unable detect values lower that zero. Induction of TGFB1 expression with a stimulant allowed us to induce TGFB1 luciferase readout such that we were able run the assay in antagonist mode. This differs from in vivo TGFB1 expression levels, which allow for evaluation of a decrease or increase in expression.

### qRT-PCR

Quantitative real time polymerase chain reaction assays were performed in triplicates (n = 3 per treatment group) in rat heart tissue derived immortalized H9C2 cells treated with cardiotoxic and reference compounds using a 384 well format on the ABI 7900HT. Relative quantification values (ΔΔCt) for *Klf4* (p/n 4331182) message were calculated using the ABI SDS 2.3 software comparing compound treatment to DMSO vehicles after normalization to *β-actin* (p/n 4352340E.) The ABI 2X Master Mix (p/n 4370074) was used with standard cycling protocols.

## Results

### Causal reasoning convergence

One of the proposed advantages in this study is the ability of the causal reasoning approach to abstract similar molecular events from microarray experiments from different sources, models and chips, thus overcoming technical and biological variability that otherwise make the comparison at the gene level challenging. Therefore, we investigated the convergence capability of CRE in detecting expected similar biological events from data generated in different species, gene-chips and different experimental settings (Iconix Drugmatrix database and GEO public data, see Methods). Isoprenaline is a widely studied prototypic compound for hypertrophic cardiomyopathy with documented molecular mechanisms [18] and its effect in rats and mice is compared here. Indeed, comparison of two independently generated gene expression datasets, for Isoprenaline treated mouse heart tissue and from rat heart tissue, reveals very similar causal reasoning biological networks (Figure 1).

The major molecular events (Figure 1) were constructed by selecting the highest ranking hypotheses and their closest significant neighbors followed by elimination of redundant and surrogate hypotheses as previously described [17]. The molecular networks from both rats and mice largely support similar biological events such as increased hypoxia/ischemia, angiotensin signaling, oxidative stress and inflammation, all of which are known mechanisms of cardiac stress response [25-29].

### Cardiac liabilities and cytotoxicity of test compounds

We selected a set of test compounds with reported ECG-type abnormalities and/or structural cardiac toxicities and of diverse pharmacology (Table 1). The ATP depletion IC50 concentration at 48 hours in H9C2 cell line was used to determine the microarray experimental concentrations. However, we harvested the cells at 24 hours for RNA extraction and microarray analysis with the rationale of investigating earlier molecular events preceding cell death. All compounds exhibited IC50 in the low micromolar range with the exception of Dexamethasone and Terbutaline.

### Examples of in vivo to in vitro causal networks

**Figure 2:**
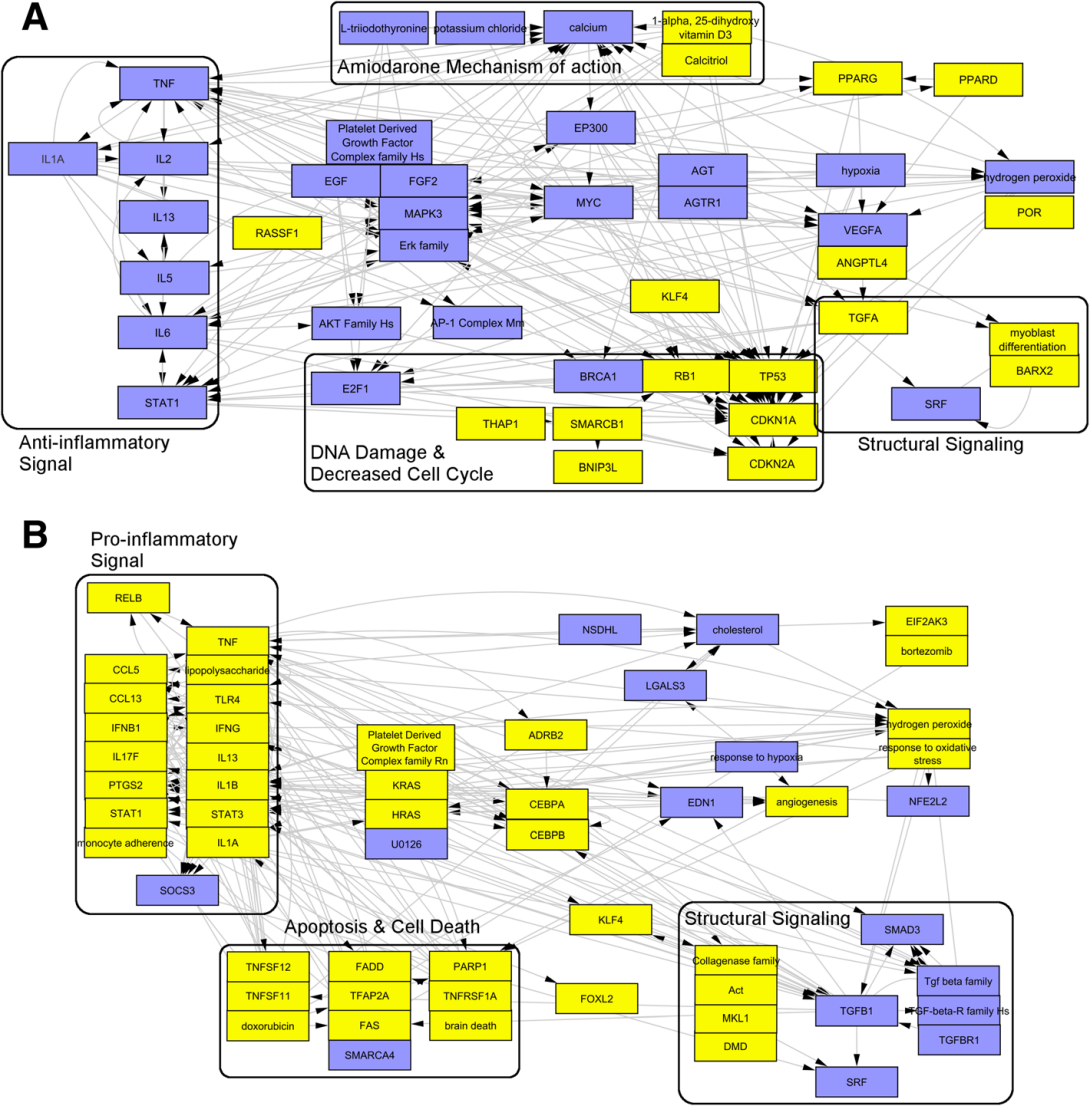
**Causal reasoning networks support poor translatability of Amiodarone induced molecular mechanisms in vivo rat heart (A) to in vitro primary rat cardiomyoctes (B).** Major differences can be seen in the lack of mechanism of action related hypotheses in vitro, predicted opposite directionality of the inflammation sub-networks, the size and composition of the tissue remodeling signal and different downstream responses (Apoptosis versus decreased cell cycle signaling). (*Blue = predicted decrease, Yellow = predicted increase*).

**Figure 3:**
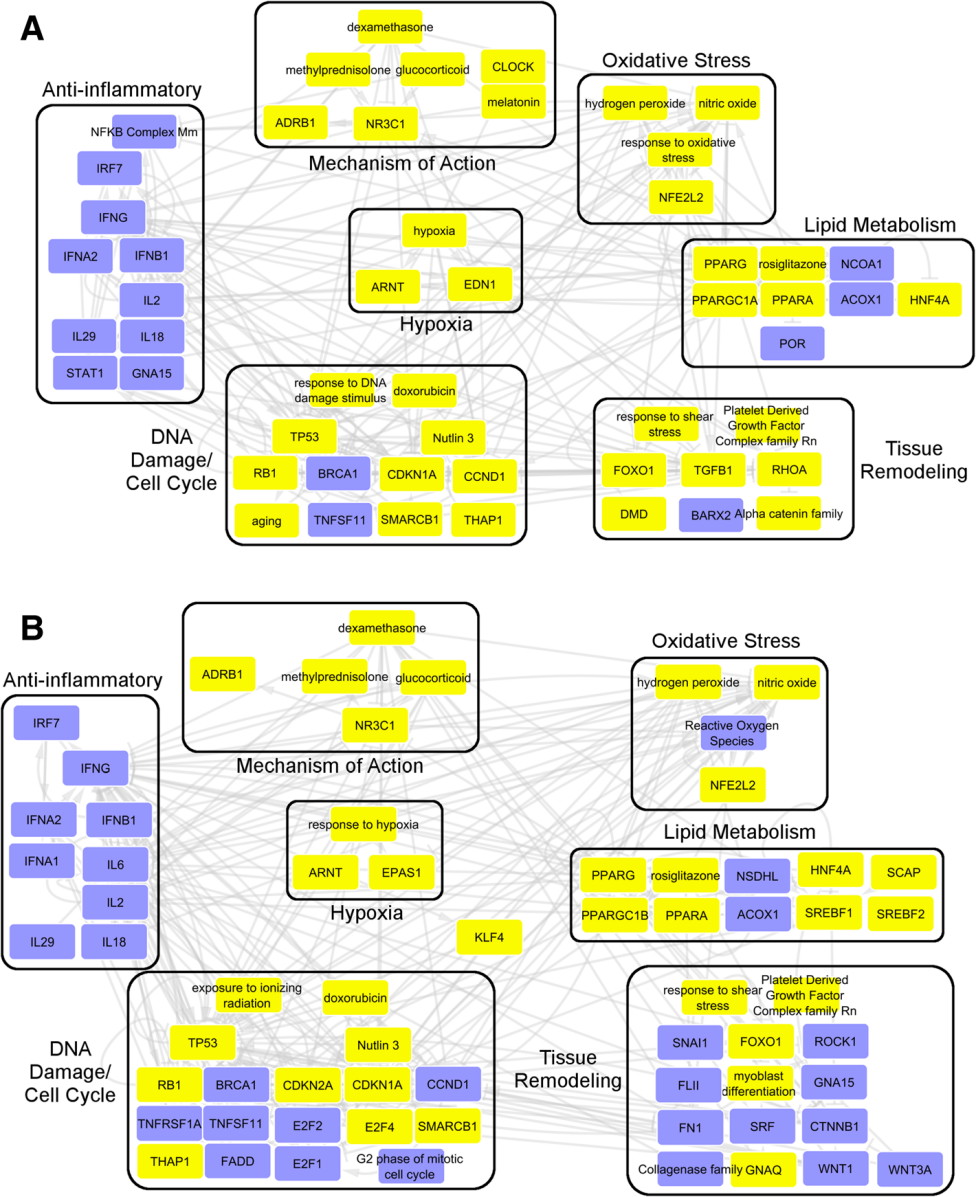
**Causal reasoning networks support high translatability of dexamethasone induced molecular mechanisms from in vivo rat heart (A) to in vitro primary rat cardiomyocytes (B).** The overview network illustrates hypotheses reflective of the mechanism of action such *Dexamethasone +* and glucocorticoid receptor *NR3C1+* as well as an anti-inflammatory hypotheses. (*Blue = predicted decrease, Yellow = predicted increase*).

All in vitro *and* in vivo experiments had a significant number of gene expression changes to drive causal reasoning analysis with the exception of Terbutaline, which did not elicit any gene expression changes in either of the two cell lines used and hence its translatability could not be further investigated. Additional file 1: Table S1 summarizes the significant CRE hypotheses and their statistical values based on the following cutoffs: 3 or more supporting genes, Enrichment and Correctness p-values <0.01 and Rank 35 or less. Figures 2 and 3 depict examples of low and high in vivo to in vitro translatability of molecular responses for Amiodarone and Dexamethasone, respectively.

Outlined in Figure 2 are the major signaling networks differentiating the Amiodarone effect on rat heart (Figure 2A) and primary rat cardiomyocytes (Figure 2B). In vivo, we found a number of hypotheses related to Amiodarone’s suggested mechanisms of action through cellular Ca^++^ and potassium modulation [30,31], and reported side effects such as binding to thyroid antagonism and hypothyroidism [32,33]. None of the mechanism related hypotheses were found in vitro. Moreover, all major causal reasoning supported biological networks were significantly different. Inflammation is one of the major signaling networks predicted, albeit with opposite directionality being predicted decreased in vivo and predicted increased in vitro. Suggested downstream effects varied significantly as well, decreased cell cycle in vivo versus apoptosis in vitro and a larger tissue remodeling/structural signal primarily driven by decreased TGFB in vitro. At the hypothesis level very few similarities were found between in vivo cardiac tissue and in vitro primary rat cardiomyoctes, e.g. *Hypoxia-* and *SRF-* hypotheses.

Contrary to Amiodarone, Dexamethasone shows high degree of in vivo to in vitro translatability at both the process and individual hypothesis levels. Figure 3 shows the causal reasoning inferred molecular response to Dexamethasone in rat cardiac tissue (Figure 3A) and Primary rat cardiomyocytes (Figure 3B). Causal reasoning generated a number of individual hypotheses reflective of dexamethasone action such as *Dexamethasone+*, *NR3C1+* and *glucocorticoid+*. Known dexamethasone effect is also reflected by supported biological processes such as the anti-inflammatory sub-network both in vivo and in vitro. Dexamethasone is also highly translatable to H9C2 cells as well with a causal network that is highly similar to that of primary rat cardiomyocytes (not shown).

### In vivo to in vitro translatability of the major biological processes

**Figure 4:**
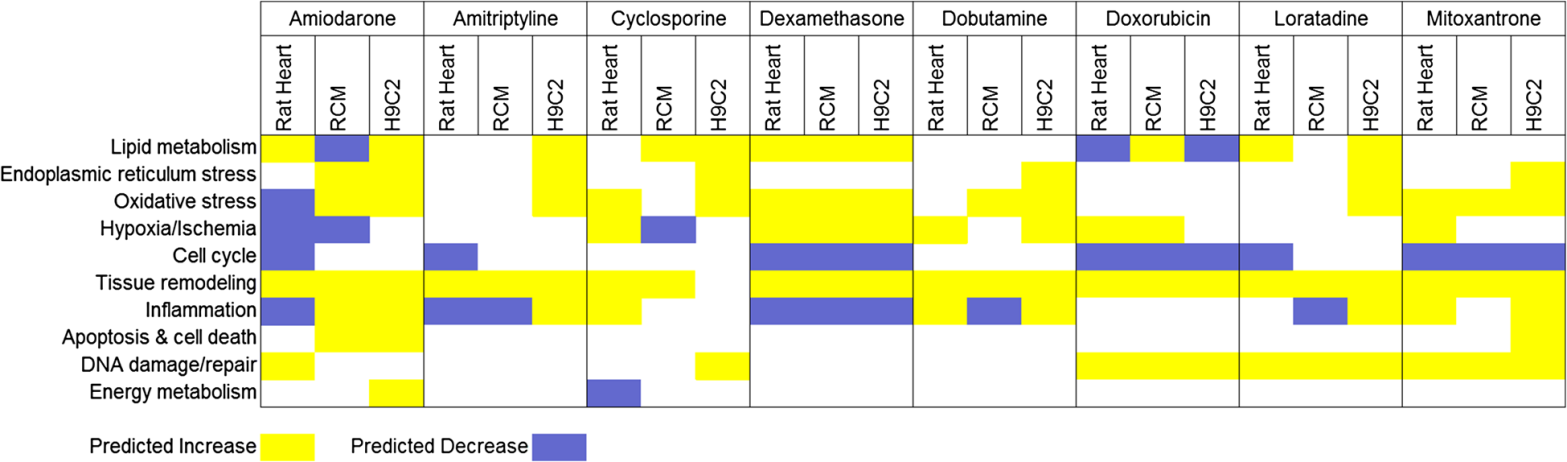
Heatmap of major causal reasoning biological networks elicited by cardiotoxic compounds in vivo and in vitro.

The top ranking causal networks from each in vivo or in vitro experiment were summarized at the biological process level in Figure 4. A network was determined to be top ranking if it was supported by a cluster of at least 3 hypotheses and one of which ranks in the top 25 hypotheses as previously described [17]. For every compound at least one process was translatable to at least one of the two cell lines used. Overall, H9C2 cells exhibited larger number of biological networks, perhaps a reflection of greater sensitivity as compared to both primary rat cardiomyocytes and in vivo cardiac tissue. H9C2 cells also demonstrated a trend of general cell stress/cytotoxicity responses that do not necessarily translate to in vivo events, such as endoplasmic reticulum stress and oxidative stress. However, for every compound there was at least one biological process that translated well from in vivo to H9C2 cells. Some of the biological processes that are supported to translate equally well in H9C2s and RCMs are decreased cell cycle signaling, increased tissue remodeling and increased DNA damage and repair. Hypoxia is one of the mechanisms that is supported to be common in vivo (6 out of 8 compounds) but does not appear to translate consistently well to neither H9C2 cells (2 out of 8 compound) nor RCMs (3 out of 8 compounds). Tissue remodeling biological processes appeared to be the most translatable across all compounds and in both H9C2s and RCMs. However, the tissue remodeling networks makeup was not necessarily homogenous in all treatments with variations in the types of hypotheses as well as the directionality of hypotheses. Examples of tissue remodeling networks included hypotheses of both increased and decreased TGFB signaling, structural protein changes such as Dystrophin (DMD) and Myocardin (MYOCD), and cytoskeleton remodeling proteins such as BARX2 and FLII.

### Identifying KLF4 as a potential common hub in cardiotoxicity

**Figure 5:**
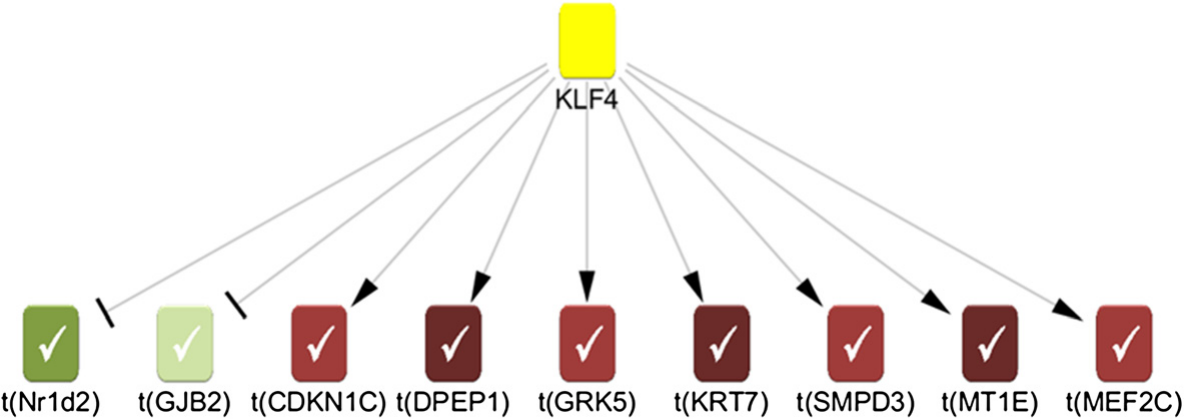
**An example of *KLF4+ *hypothesis subgraph showing the observed gene expression changes that led to the predicted increase of activity in in vitro H9C2 cells.** Yellow = predicted increase, Red = observed mRNA increase and Green = observed mRNA decrease.

**Figure 6:**
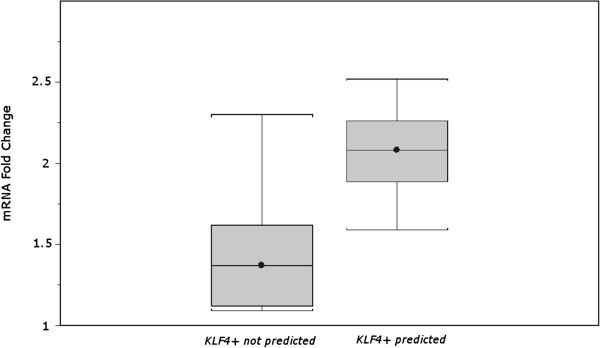
**Box plot shows a trend where predicted *KLF4 *hypothesis had a corresponding observed increase in *KLF4 *mRNA greater than 1.5 fold on the gene chip for in vitro H9C2 cells.** However, CRE did not predict all significant increases of *KLF4* mRNA.

*KLF4+* was one of the frequent hypotheses in both cell lines and in vivo (See Additional file 2: Figure S1). Additionally, *KLF4+* was found to be connected to key hypotheses from different toxicity mechanisms such as *IFNG* in inflammation, *TGFB1* in tissue remodeling and *TP53* and *CDKN1A* in cell cycle (see example networks in Figures 2 and 3). This suggests a potential role of KLF4 as a central hub in cardiotoxicity. Figure 5 shows an example of a *KLF4+* hypothesis and the supporting observed gene expression changes. In addition to the CRE prediction of increased KLF4 activity the observed KLF4 gene expression levels from the Affymetrix gene chips showed consistent increase correlating well with the CRE predictions (Figure 6). Finally, subsequent follow-up RT-PCR experiment to measure KLF4 mRNA in H9C2 in response to treatment showed consistent results (Table 2). Doxorubicin was one of the exceptions where there was observed decrease in mRNA on the Affymetrix gene chip despite of predicted *KLF4+* hypothesis. However, repeating the experiment with a lower Doxorubicin concentration that corresponds to the IC20 resulted in 2.52 fold increase in *KLF4* mRNA perhaps suggests the CRE prediction was for a molecular event at an earlier time point.

**Table 2:** ***KLF4 *mRNA fold changes in H9C2 from the gene microarrays and subsequent confirmation using qRT-PCR**

	**qRT-PCR**	**Affymetrix**
Amiodarone	2.2	1.62
Amitryptiline	2.8	2.3
Cyclosporine	2.1	1.84
Dexamethasone	1.3	2.14
Dobutamine	1.7	1.93
Doxorubicin	−9.7*	−4.92*
Loratadine	2.4	2.1
Mitoxantrone	1.4	2.06

*The decreased levels in Doxorubicin might be a reflection of severe cytotoxicity. However, *KLF4* measurement at a lower dose (IC20 instead of IC50) resulted in 2.52 fold increase.

### Potential role of TGFB1 in cardiotoxicity and TGFB1 reporter assay

**Figure 7:**
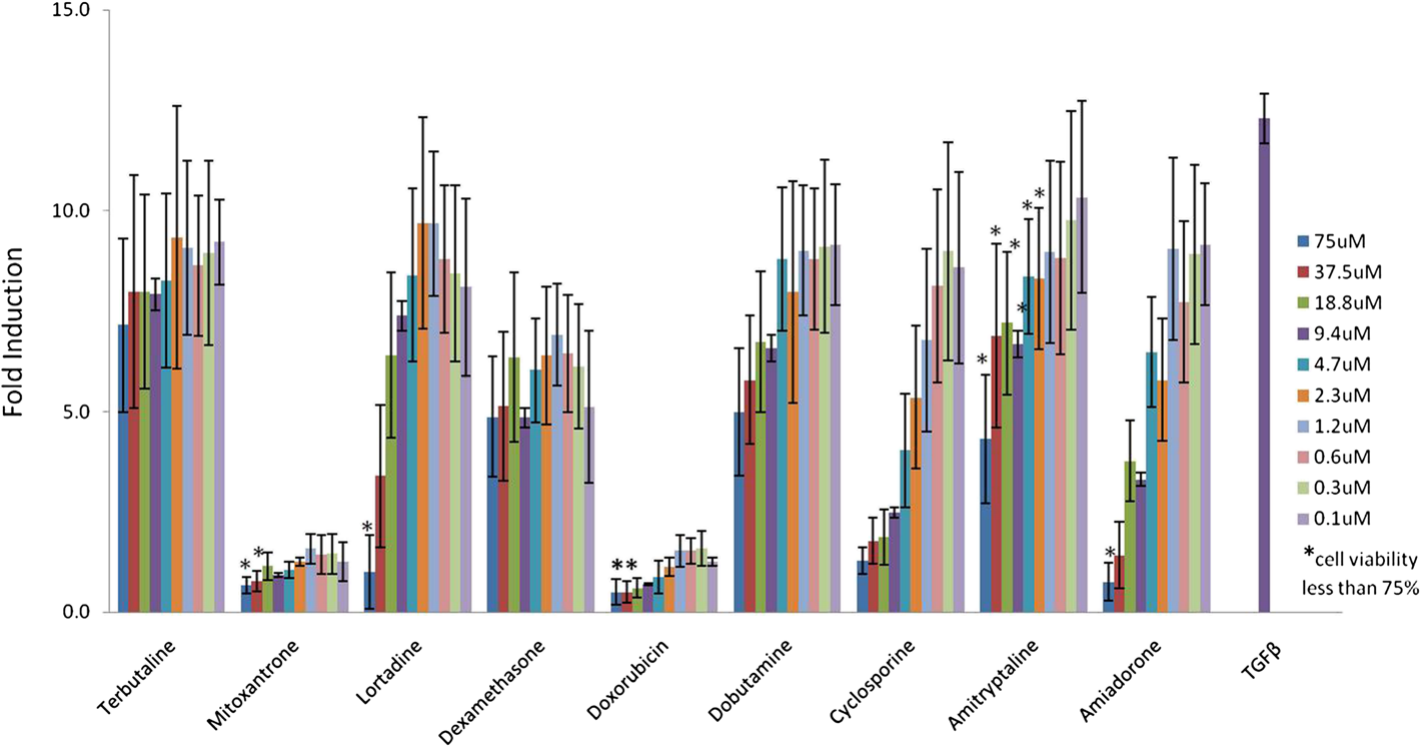
**Inhibition of TGFB reporter assay.** HEK293-TGFB reporter cells treated in dose response with compounds known to induce cardiac toxicity.

TGFB signaling was one of the most frequently perturbed signaling pathway in vivo and in vitro with all tested compounds with the exceptions of Dexamethasone in RCM and Cyclosporine in H9C2 cells. However, the perturbation was in many cases in opposing directions in vivo vs. in vitro (Table 3). Next, we employed a TGFB1 reporter assay to experimentally test the predicted effect of compounds on TGFB1 activity in vitro. Compound treatment following stimulation with TGFB1 demonstrates the inhibitory effect of the compounds in dose dependant manner consistent with the CRE predictions (Figure 7). In absence of TGFB1 stimulation none of the tested compounds had a stimulatory effect (data not shown).

**Table 3:** Predicted TGFB signaling in vivo and in cell lines

	**Predicted TGFB signaling**		
	**In vivo**	**H9C2**	**RCM**
Amiodarone	↑	↓	↓
Amitryptiline	↑	↓	↓
Cyclosporine	↑	↔	↓
Dexamethasone	↑	↑	↔
Dobutamine	↑	↓	↓
Doxorubicin	↑	↑	↓
Loratadine	↑	↓	↓
Mitoxantrone	↑	↓	↓

## Discussion

Gene expression changes of nine compounds known to induce cardiotoxicity were profiled in rat cardiomyocytes, rat embryonic heart tissue-derived H9C2 cells, and heart tissue from treated rats. There was, as expected, significant variation between drugs and test systems at the individual gene level. In this work we applied a recently developed method [16,17] to understand convergence of gene expression changes based on their potential upstream regulators. As described the CRE analysis revealed a convergence of the explained changes around a set of biological pathways. Specifically, pathways associated with tissue remodeling, cell cycle, oxidative stress, and DNA damage were particularly well conserved across cardiotoxic drugs and between in vivo and in vitro test systems. This level of concordance between the in vivo and in vitro systems was encouraging but there were some clear points of disagreement between the experimental systems providing a stark reminder of the limitations of in vitro systems. An example of this difference is the greater diversity of signaling in H9C2 cells compared to rat cardiomyocytes. This may be explained by the immortalized nature of H9C2 cells with active cell cycle compared to the primary rat cardiomyocytes. Another possibility is that H9C2 cells are less similar to cardiomyocytes thus more likely to exhibit non-cardiomyocyte phenotype. Although, the wholesale differences between the Amiodarone in vitro and in vivo transcriptional changes highlights that the overall predictivity of cellular systems can vary from compound to compound depending on specific expression of drug targets, the opposing TGFB signals observed across the majority of tested drugs points to a more fundamental inability of the in vitro systems to replicate in vivo signaling networks. By better understanding these limitations though, we might still be able to address those instances of successful translations of pathway-level signals of toxicity between in vivo and in vitro systems to quickly and efficiently triage potential therapeutics for their potential to induce adverse events.

The CRE method provided interesting insights in this case and summarized the observed expression changes efficiently for further analysis. However, it is important to note its potential shortcomings. The approach is only as powerful as its underlying knowledgebase of prior biological knowledge. Even a knowledgebase that encompasses all currently known biomedical relationships would not be able to summarize changes that have never been observed before. In our experience [17] the approach usually provides helpful insights as many molecular regulatory processes have been well researched over time. Given a comprehensive knowledgebase results often turn up combinations of upstream regulators that have been observed in a different biological context previously but are novel for the biological problem under study.

Almost as important as the overlap between the in vivo and in vitro outcomes of drug treatment is the notion that the critical biological processes that seem to underlie the drug toxicity can be visualized across various cell types. Much work has been devoted to trying to build an in vitro system that accurately replicates intact organ systems in a dish. These technologies have tended to be expensive, laborious, and low-throughput, thus limiting their utility in any type of routine predictive screening strategy. In addition, these complex culture systems still fail to fully recapitulate the in vivo organ system they seek to model, particularly for long term dosing studies. What this work suggests though is that these types of convoluted cell models might not be necessary for understanding the safety risk of a segment of compounds. When the underlying mechanism of the toxicity is a basic pathway associated with cell health and viability, the specific cell system is of minimal importance. Moving from a primary cardiomyocyte, which recapitulates many important activities of an in vivo cardiac cell; to an immortalized rat heart tissue derived cell line such as H9C2 did not result in the loss of translational power. Likewise, the primary cardiomyocytes were just as likely to show discordance from the in vivo as the immortalized cell line was.

The traditional thinking has been that the reason for the organ specificity of drug toxicity was due to unique innate traits of the particular organ being affected. This thinking has largely driven a desire to have more organ-like in vitro culture systems. The notion that very generic, non-organ specific mechanisms of toxicity might explain a large portion of organ-specific toxicity runs counter to this thinking and leads to questions of why compounds with these types of liabilities do not show gross, multi-organ toxicities in vivo. It has long been appreciated that differences in distribution and accumulation of medications directly affect their efficacy [34]. The same can be said about toxicity. Cardiotoxicity is not entirely due to the unique “cardiac-ness” of the cells but due to the fact that the heart is the organ that sees the greatest concentration of the compound as a result of a combination of intrinsic and extrinsic expression of transporters and clearance mechanisms. Therefore, in an in vitro system, where one can ensure exposure of the compound to the cell, reproducing an intact organ system is not necessary for visualizing the toxicity risk.

This is not to say that all types of toxicity can be modeled in a generic cell line. There are several types of specific drug-induced toxicities were specific functionalities must be present in a cell system in order to visualize that toxicity. For example, induced pluripotent stem cell derived cardiomyocytes have been extensively characterized (including comparative gene expression profiling) and evaluated to study cardiac specific end points (such as Beating and Contractility) [35,36]. Utilization of these types of advanced test systems that take advantage of ‘cardiac-ness’ of these cells might be helpful for certain evaluations. This may be the case for Amiodarone in this study. For instance, drug-induced arrhythmias could be attributed to a very unique feature of cardiomyocytes. Ideally, an in vitro system that predicts this outcome would incorporate a cell that beats so that any alteration in pace or occurrence of rhythmic cell contraction could be directly measured. But even with this example, distilling this very organ-specific toxicity down to the basic molecular mechanism that drives it enables a simple, cell-neutral assay for predicting it, hERG binding and dofetilide competition. As we gain a better appreciation of the mechanisms of toxicity, there will be a reduction in the need for costly primary cell cultures in predictive toxicology.

The mechanisms of toxicity uncovered in this work are not entirely novel. Disregulating cell cycle, inducing DNA damage, and producing oxidative stress has long been appreciated as having a negative effect on cellular health, often leading to obvious cytotoxicity. It is not surprising then that a basic cytotoxicity assay has been shown to have high predictive power for in vivo toxicity regardless of the organ-specific nature of that toxicity [37,38]. This similarity in toxicity across cell lines of different tissue origins can also be seen in our data. Both the primary cardiomyocytes and immortalized skeletal muscle cells showed a clear down regulation of TGFB signaling upon application of cardiotoxicants. We were able to reproduce this data utilizing a reporter system cloned in cell line derived from kidney. Although this response was in opposition to what was observed in vivo, upon moving to the in vitro system, there was a complete conservation of signaling at the pathway level regardless of the tissue type the cell line was meant to model. Similarly, both primary cardiomyoctes and immortalized H9C2 cells showed predicted increase in KLF4 which we were able to reproduce by measuring KLF4 expression levels using RT-PCR. KLF4 is a hub that mediates the effect of different cell stress signals such as oxidative stress and DNA damage on critical cell functions such as cell proliferation and differentiation [39]. In particular, KLF4 is known to play a role in cardiac function. For example, KLF4 has been shown to mediate cardiac myofibroblast differentiation in response to Angiotensin II stimulation partly through regulating TGFB1 [40]. KLF4 has been also shown to be involved in regulating the cardiac hypertrophic response [41].

The finding concerning TGFB signaling has implications beyond this work. In recognition of the need for more and better in vitro tools for toxicity prediction, many different reporter assays and screening systems have been built and are being marketed for this purpose. The choice of signaling pathways and cellular endpoints used for these products are, for the most part, based not on detailed validation of the tools for their designed purpose. Instead the significance of these endpoints is taken exclusively from literature without fully understanding the impact of moving them to an in vitro detection system. The link between aberrant TGFB signaling and potential adverse events is well established [42-44]. Using a reporter system to measure the potential of a compound to induce that signaling network in vivo is clearly not that straightforward though, based on the finding of this work. Until the translatability of tools like the TGFB reporter system can be validated, caution must be taken in utilizing it and tools like it for predictive screening.

## Conclusions

There is a desperate need in modern drug discovery for high-throughput, cost effective assay technologies that are highly predictive of in vivo toxicity. One of the primary concerns in adapting these assays for triaging newly developed compounds is the ability to translate an in vitro signal to an in vivo outcome. This work adds to the growing literature that strongly suggests that an in vivo/in vitro connection can be drawn through the use of basic cellular mechanisms but there are limitations to these predictions that are independent of the relationship between the cell type and the target tissue.

## Competing interests

The authors declare that they have no competing interests.

## Financial competing interests

In the past five years have you received reimbursements, fees, funding, or salary from an organization that may in any way gain or lose financially from the publication of this manuscript, either now or in the future? Is such an organization financing this manuscript (including the article-processing charge)? **No**

Do you hold any stocks or shares in an organization that may in any way gain or lose financially from the publication of this manuscript, either now or in the future? **No**

Do you hold or are you currently applying for any patents relating to the content of the manuscript? Have you received reimbursements, fees, funding, or salary from an organization that holds or has applied for patents relating to the content of the manuscript? **No**

Do you have any other financial competing interests? **No**

## Non-financial competing interests

Are there any non-financial competing interests (political, personal, religious, ideological, academic, intellectual, commercial or any other) to declare in relation to this manuscript? **No**

## Authors’ contributions

AEE carried study conception, experimental design, data analysis & interpretation and manuscript writing, DP contributed to experimental design, execution and manuscript writing, DZ contributed Data analysis and manuscript writing, JEF carried experimental execution and manuscript writing, SK contributed to experimental execution and manuscript writing, MTP contributed to study conception, experimental design, interpretation and manuscript writing. All authors read and approved the final manuscript.

## Pre-publication history

The pre-publication history for this paper can be accessed here:

http://www.biomedcentral.com/2050-6511/14/46/prepub

## Supplementary Material

Additional file 1: Table S1 Summary of the statistical values used to determine significance of the CRE hypotheses. These include ‘Enrichment’ and ‘Correctness’ p-values, number of ‘Correctness’ genes and the “Rank” of hypotheses based on the on the difference between enriched genes supporting the ‘Correctness’ and enriched genes not supporting the ‘Correctness’. Click here for file

Additional file 2: Figure S1 Scatter plot analysis for KLF4 transcript levels measured by Affymetrix microarray and RT-PCR. The fold change cut-off (1.3) used for the CRE analysis is indicated by the horizontal and vertical lines. Click here for file
