# Supplementary figures and images for: Assessing the translatability of In vivo cardiotoxicity mechanisms to In vitro models using causal reasoning
Source: BMC Pharmacol Toxicol. 2013 Sep 6;14:46. doi: 10.1186/2050-6511-14-46 (PMC3846863; doi:10.1186/2050-6511-14-46)

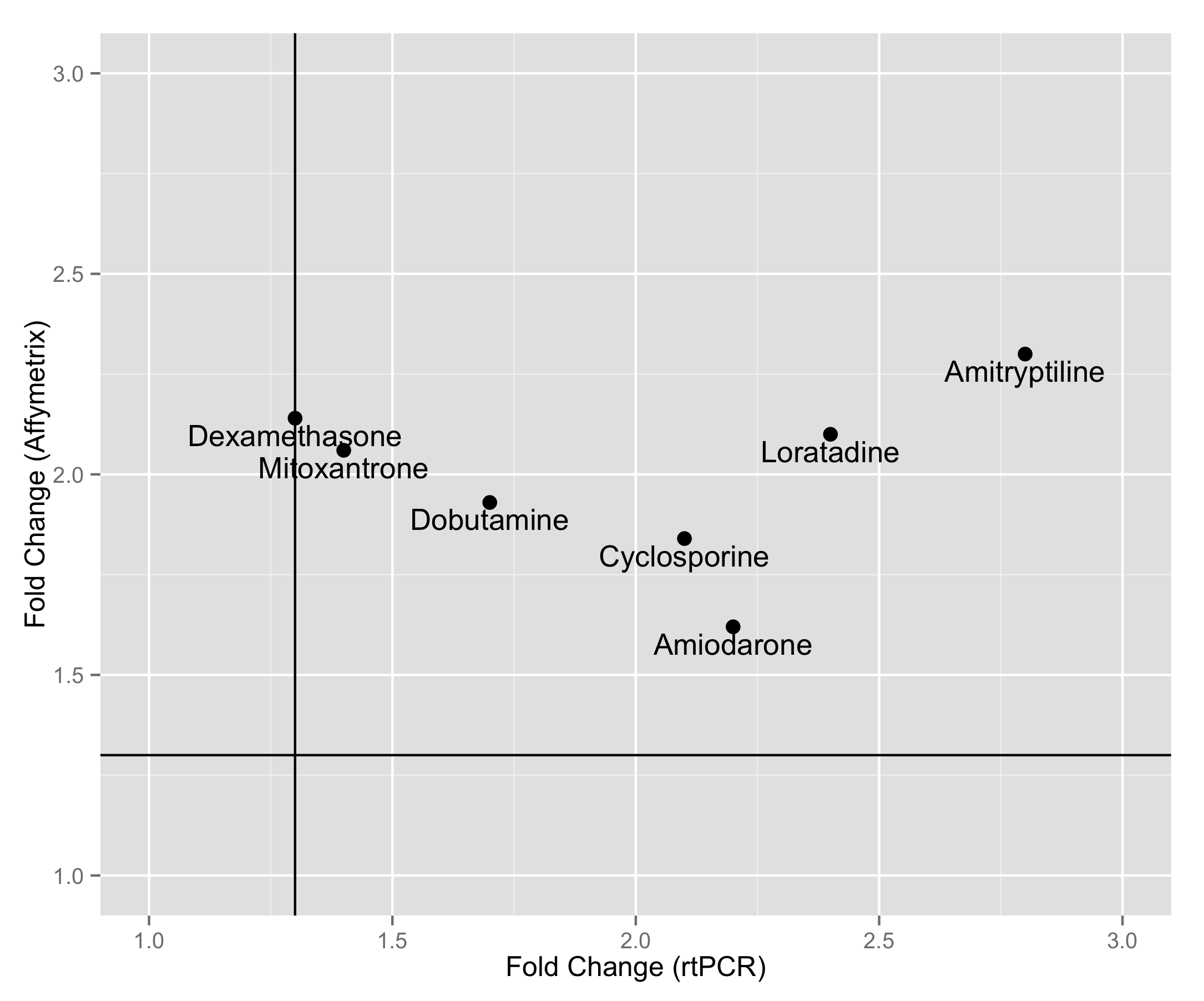

Supplement: Additional file 2: Figure S1 — Scatter plot analysis for KLF4 transcript levels measured by Affymetrix microarray and RT-PCR. The fold change cut-off (1.3) used for the CRE analysis is indicated by the horizontal and vertical lines. [file 2050-6511-14-46-S2.tiff]
